# Telemedicine vs Telephone Consultations and Medication Prescribing Errors Among Referring Physicians: A Cluster Randomized Crossover Trial

**DOI:** 10.1001/jamanetworkopen.2024.0275

**Published:** 2024-02-29

**Authors:** James P. Marcin, Monica K. Lieng, Jamie Mouzoon, Hadley S. Sauers-Ford, Daniel Tancredi, Annie Cabri, Vaibhavi A. Pandya, Alex S. Park, Nathan Kuppermann

**Affiliations:** 1 Department of Pediatrics, University of California School of Medicine, Davis Health, Sacramento; 2 Department of Emergency Medicine, University of California, Davis Health, Sacramento; 3 Department of Pharmacy, University of California, Davis Health, Sacramento

## Abstract

**Importance:**

Critically ill children presenting to emergency departments (EDs) in non–children’s hospitals are at high risk for experiencing medical errors, including medication errors. Video telemedicine consultations with pediatric specialists have the potential to reduce the risk of medication errors beyond the current standard of care, telephone consultations.

**Objective:**

To compare the rates of ED physician-related medication errors among critically ill children randomized to receive either video telemedicine or telephone consultations.

**Design, Setting, and Participants:**

This cluster randomized, unbalanced crossover trial was conducted at 15 community EDs in northern California between September 2014 and March 2018. Analyses were conducted from May 2022 to January 2023. Participants included acutely ill children younger than 15 years presenting to a participating ED.

**Interventions:**

Participating EDs were randomized to use video telemedicine or telephone for consultations with pediatric critical care physicians according to 1 of 4 unbalanced (3 telemedicine to 1 telephone) crossover treatment assignment sequences.

**Main Outcomes and Measures:**

Pharmacists reviewed medical records to document physician-related medication errors using a previously validated instrument. Multilevel logistic regression analyses were performed to create models with the medication order as the unit of analysis and adjusting for age, the log-transformed Revised Pediatric Emergency Assessment Tool score, and hospital study period.

**Results:**

A total of 696 patient encounters were included in the trial (mean [SD] age, 4.2 [4.6] years; median [IQR] age, 2.1 [0.5-2.1] years; 304 female [43.7%]), with 537 patient encounters (77.2%) assigned to video telemedicine and 159 patient encounters (22.8%) assigned to telephone. At least 1 physician-related medication error occurred for 87 patients (12.5%), including 20 of 159 patients (12.6%) in the telephone cohort and 67 of 537 patients (12.5%) in the telemedicine cohort. Of the 2414 medication orders, errors occurred in 124 cases (5.1%), including 26 of 513 orders (5.1%) in the telephone cohort and 98 of 1901 orders (5.2%) in the telemedicine cohort. In the multivariable analysis, the adjusted odds ratio of experiencing a medication error among those assigned to telemedicine was 0.86 (95% CI, 0.49-1.52; *P* = .61).

**Conclusions and Relevance:**

This cluster randomized crossover trial found no statistically significant differences in physician-related medication errors between critically ill children assigned to receive telephone consultations vs video telemedicine consultations.

**Trial Registration:**

ClinicalTrials.gov Identifier: NCT02877810

## Key Points

**Question:**

Does the use of video telemedicine for pediatric consultations to referring hospital emergency departments (EDs) result in less frequent medication errors than the current standard of care, telephone consultations?

**Findings:**

In this cluster randomized crossover trial that included 696 acutely ill children presenting to 15 community and rural EDs, there were no statistically significant differences in physician-related medication errors between children assigned to receive telephone consultations vs video telemedicine consultations.

**Meaning:**

These findings suggest that the use of video telemedicine to conduct consultations for acutely ill children in rural and community EDs does not result in less frequent medication errors than consultations done by telephone.

## Teaser

This cluster randomized crossover trial compares the rates of emergency department physician–related medication errors among critically ill children randomized to receive either video telemedicine or telephone consultations.

## Introduction

Medication errors are a leading preventable cause of adverse events in health care.^1^ Patients presenting to emergency departments (EDs) are at high risk of medication errors for several reasons, including that EDs are traditionally chaotic environments^2,3^ and often lack access to pharmacist review.^4^ Children are at even higher risk, up to 3 to 4 times more likely than adults to experience a medication error during an ED encounter.^4,5^ Children require weight-based dosing, and because of relatively low volumes of children evaluated in non–children’s hospital EDs, practitioners may be less familiar and less confident in ordering medications for children.^6,7^ One study^8^ found that 39% of medication orders from encounters with critically ill children presenting to rural EDs in northern California had at least 1 medication error.

Consultation with a pediatric specialist by either telephone or video telemedicine has the potential to decrease the frequency of medication errors, given that pediatric specialists are more experienced in pediatric prescribing. A nonrandomized study^9^ of 8 critical access hospitals found that pediatric ED encounters with telemedicine consultations were associated with fewer medication errors than ED encounters with telephone consultations and ED encounters with no consultations. To our knowledge, there have been no randomized trials evaluating whether telemedicine consultations provide additional benefit over telephone consultations with respect to physician-related medication errors in community EDs. To address this gap, we conducted a large, multi-institutional, 2-year randomized trial to determine the impact of pediatric video telemedicine emergency consultations compared with telephone consultations on physician-related medication errors. We hypothesized that critically ill children who received care in these EDs during periods where video telemedicine consultations were assigned would experience fewer physician-related medication errors than children who received care during periods where telephone consultations were assigned.

## Methods

### Study Design and Participating Hospitals

We sought to conduct a pragmatic trial, conducted in a clinical practice setting and with broad patient inclusion criteria and clinicians, designed to evaluate the impact of telemedicine consultations on physician-related medication errors in a robust clinical trial.^10^ The resulting trial design was a pragmatic, cluster randomized trial that used an unbalanced crossover design in which EDs, the units of randomization, were stratified by size and geographic location. This study received institutional review board approval from University of California, Davis. We followed the Consolidated Standards of Reporting Trials (CONSORT) reporting guideline for randomized clinical trials. The need for informed consent was waived because the patients’ data were deidentified, in accordance with 45 CFR §46. The study protocol is shown in Supplement 1.

From the more than 30 hospital EDs in the University of California Davis Children’s Hospital (UCDCH) catchment area with video telemedicine capabilities, we selected 16 EDs to ensure a diverse sample. These included 6 critical access hospitals and 12 hospitals located in Health Resources and Services Administration–designated rural geographic areas. Eight of the EDs had access to on-call consulting pediatricians, but none of the hospitals had local pediatric hospitalist physicians, pediatric emergency medicine physicians, or pediatric critical care physicians. All EDs had electronic health records that included computerized physician order entry (CPOE).

We chose a crossover design over a parallel group design (in which each ED would be randomized to either telemedicine or telephone for the entire duration of the study) for methodological, ethical, and statistical reasons. By assigning EDs to 1 of 4 period sequences where each sequence contained 3 periods assigned to telemedicine consultation and 1 period assigned to telephone consultation, the study design allows each ED to serve as its own control. This required a smaller sample size than a parallel cluster randomized trial would need, while also avoiding the ethical hurdle that could arise from assigning some EDs to a telephone-only group. We choose a 3:1 unbalanced allocation of the study periods because, from previous experience and because of the additional workflow burden, we anticipated that when EDs were in periods assigned to video telemedicine, there would still be a high rate of telephone use.

The 16 hospitals were binned into 4 strata. Within each stratum, EDs were randomized into 1 of the four 4-period sequences (ie, schedules) that covered an enrollment period of 2 years for each hospital. In each schedule, a letter refers to a 6-month block. With T representing video telemedicine time blocks and P representing telephone time blocks, the 4 possible schedules were PTTT, TPTT, TTPT, or TTTP. To remind and encourage consulting physicians to use the assigned modality, interfacility transfer request cards, used to document all consultation requests, included participating ED names and modality assignments. In addition, as a nudge strategy, emails were sent to each consulting physician following every consultation and were shared at weekly physician meetings with protocol adherence rates for all physicians. These efforts were meant to encourage the use of the assigned modality throughout the trial.

### Patient Population

Children were included in the study if they were younger than 15 years and if the treating emergency medicine physician placed a telephone call to the UCDCH transfer center to request a consultation from a pediatric critical care physician and/or request to transfer to the pediatric intensive care unit. The age of 15 years was selected as a threshold to reduce potential bias introduced by the fact that, in some instances, children aged 16 years and older are routed to adult critical care physicians. Children with acute injuries were routed to the pediatric trauma surgeons and were not included in this study.

### Video Telemedicine Intervention

Video telemedicine units consisted of pole-mounted, high-resolution video conferencing units with remote pan-tilt-zoom capabilities. All consultations were initiated by a telephone call from the outside ED to the UCDCH transfer center to connect to a UCDCH pediatric critical care physician. During the telemedicine-assigned periods, the UCDCH pediatric critical care physicians were encouraged to complete the consultations over video telemedicine. During the telephone-assigned consultations, the physicians were encouraged to complete the consultations over the telephone. Although physicians were made aware of the assigned consultation modality, deviations were permitted, and the consultation modality was ultimately determined by agreement between physicians. In neither case did the UCDCH pediatric critical care physicians have access to the electronic health records at the participating ED. Pediatric critical care physicians could initiate the telemedicine call at a workstation in the UCDCH pediatric intensive care unit. Video telemedicine consultations typically involved some combination of the treating physician, nurses, and respiratory therapists in addition to the patient and parent or guardian (when present). More details on the technological specifications of the video telemedicine setup and integration into workflow have been reported elsewhere.^11^

### Sample Size

Our trial was designed to satisfy several objectives, including assessing impacts on medication errors. From previous experience conducting pediatric telemedicine emergency consultations, we expected approximately 2% of all pediatric ED encounters at participating EDs to result in pediatric specialty consultation, and 25% of these to receive a pediatric critical care consultation. Sample size calculations were determined for a previously reported outcome of this study, interfacility transfer rates.^11^ The planned enrollment of at least 448 patients provided at least 80% power to detect a clinically important adjusted odds ratio of 0.3 for the medication error outcome. This calculation assumed that approximately 10% of medications during the telephone-only periods would result in a physician-related error (defined later), on the basis of previously reported error incidences.^8,9^ Hence, an odds ratio of 0.3 applied to an incidence of 10% results in an incidence lowered to 3.2%.

### Study Outcomes: Medication Errors

Medication errors were defined using the definitions and taxonomy from the National Coordinating Council for Medication Error Reporting and Prevention.^12^ We used Lexi-Comp, Inc, guidelines^13^ for appropriate medication dosing ranges. From these published definitions and guidelines, a medication error evaluation instrument was developed and validated by our research group.^8^ We focused on physician-related ED medication errors because we hypothesized that telemedicine and telephone consultations would most likely affect emergency medicine physician prescribing rather than administration and delivery of the medication. Physician-related ED medication errors were defined as those involving a wrong dose, defined as between 10% and 25% of appropriate dose, a wrong or inappropriate medication for the patient’s condition, a wrong route of administration, a wrong dosage form, or errors regarding patient information, such as a known allergy.^8,9^ The instrument was applied to all patient records and was used to evaluate all medication orders, including prescription and nonprescription medications. The data collection instrument and guidelines used to determine medication errors are provided in the eAppendix in Supplement 2.

### Data Collection

Hospitals were enrolled for 2 years, with staggered start dates between September 2014 to March 2016 and end dates between September 2016 and March 2018. Deidentified electronic medical records were obtained from the originating EDs with any direct and indirect references to the type of consultation (telephone or video telemedicine) redacted. Demographic data and encounter characteristics, including use of emergency medical services for transportation to the ED, were entered into a REDCap database.^14^ Severity of illness was measured using the validated Revised Pediatric Emergency Assessment Tool.^15^ The entire medical record from the ED visit, including physician documentation, vital signs, nursing notes, laboratory results, medication orders, and other records, were available for pharmacist review. Three pediatric pharmacists (A.C. V.A.P., and A. S.P.), who were not involved in the patient’s care, independently determined medication errors and assigned error severity using the redacted electronic medical records; 90% of the records were reviewed by 1 of the 3 pediatric pharmacists. To estimate interrater reliability, 10% of patient records were reviewed by all 3 pediatric pharmacists.

### Statistical Analysis

Analyses were conducted from May 2022 to January 2023. We used Pearson χ^2^ test to perform bivariable comparisons of the 2 consultation modes for categorical variables and the Wilcoxon-Mann-Whitney test for continuous, nonnormal variables, with significance set at 2-sided *P* < .05. The units of analysis for this study were individual medications, which were nested within patients seen in the originating EDs. We used 3-level mixed-effects logistic regression with random intercepts for patient and for originating ED. For the patients rated by the 3 pediatric pharmacists, we fit a mixed-effects model with separate random intercepts for originating EDs, patients, and medication and used the sum of the variance components for these terms relative to the sum of all 4 variance components (including residual error) to estimate interrater reliability. Covariates were chosen a priori and included patient age, the natural log-transformed severity of illness as measured by Revised Pediatric Emergency Assessment Tool, and hospital time-period in hospital (eg, first, second, third, and fourth) to adjust for secular trends. Because this was a pragmatic trial, we anticipated deviations in the treatment groups and report intention-to-treat, treatment-received, and per-protocol analyses. Statistical analyses were conducted in R statistical software version 3.6.1 (R Project for Statistical Computing) and Stata statistical software version 15 (StataCorp).

## Results

### Characteristics of Study Sample

A total of 696 patient encounters were included in the trial (mean [SD] age, 4.2 [4.6] years; median [IQR] age, 2.1 [0.5-2.1] years; 304 [43.7%] female), with 537 patient encounters (77.2%) assigned to video telemedicine and 159 patient encounters (22.8%) assigned to telephone (Figure). These encounters came from 15 EDs, because 1 of the participating hospitals closed shortly after the start of the trial. As a pragmatic trial, 309 of the 537 encounters (57.5%) assigned to video telemedicine received telephone consultations and 23 of the 159 encounters (14.5%) assigned to telephone received video telemedicine consultations. Consultations occurred approximately 2.5 hours after the patient presented to the ED. Baseline and demographic variables were similar for the telemedicine and telephone groups in the intention-to-treat analyses (Table 1).

**Figure.**
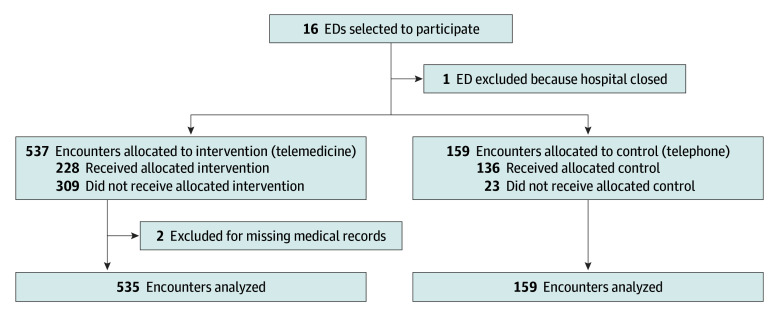
Emergency Department (ED) Enrollment Flowchart

**Table 1.** Baseline Characteristics by Study Allocation (Intention-to-Treat)

Characteristic	Patients or encounters, No. (%)	
	Telephone (n = 159)	Video telemedicine (n = 537)
Patient characteristics		
Age, median (IQR), y	1.63 (0.24-5.59)	2.33 (0.54-7.67)
Sex		
Female	73 (45.9)	231 (43.0)
Male	86 (54.1)	306 (57.0)
Insurance status		
Private	39 (24.5)	105 (19.6)
Medi-Cal, self-pay, no insurance, or other	120 (75.5)	432 (81.4)
Encounter characteristics		
Consultation received		
Telephone	136 (85.5)	309 (57.5)
Telemedicine	23 (14.5)	228 (42.5)
Emergency medical services use	33 (20.8)	131 (24.4)
Revised Pediatric Emergency Assessment Tool score, median (IQR)	1.61 (1.14-2.06)	1.54 (1.09-2.02)
Time to consultation, median (IQR), h	2.4 (1.4-3.9)	2.5 (1.2-3.9)
Medication error characteristics		
No.	159	535
Patients with medication orders	130 (81.8)	423 (78.8)
Medications per patient, No.		
Mean (SD)	3.2 (3.2)	3.6 (3.0)
Median (IQR)	2.0 (1.0-4.0)	3.0 (1.0-5.0)
Errors per medication, mean (SD)	0.12 (0.27)	0.10 (0.25)
Medication errors per patient, median (IQR)	0.0 (0.0-0.5)	0.0 (0.0-0.0)
Patients with 0 medication errors	139 (87.4)	468 (87.5)
Patients with 1 medication error	15 (9.4)	50 (9.3)
Patients with ≥2 medication errors	5 (3.1)	17 (3.2)
Patients with ≥1 medication error	20 (12.6)	67 (12.5)

### Physician-Related Medication Errors

Two encounters from the medication error analyses that were assigned to the video telemedicine consultation cohort were excluded because medication data were not able to be extracted from the electronic health record owing to a loss of electronic health record data (Figure). Of the 694 remaining encounters, 159 patients in the telephone cohort were prescribed a mean (SD) of 3.2 (3.2) medications, and 535 patients in the telemedicine cohort were prescribed a mean (SD) of 3.6 (3.0) medications. The interrater reliability among pharmacist reviewers for a physician-related error for each medication was moderate at 0.48 (95% CI, 0.27-0.71). Most patient encounters (607 patients [87.5%]) did not experience a physician-related medication error; 67 patients (9.7%) experienced a single medication error, and 20 patients (2.8%) had 2 or more medication errors. As shown in Table 1, there were 20 patients in the telephone cohort who experienced at least 1 medication error (12.6%) and 67 patients in the video telemedicine cohort who experienced at least 1 medication error (total, 87 errors [12.5%]). There were a total of 2414 medication orders and 124 errors (5.1%); 26 errors were identified among the 513 medication orders in the telephone cohort (5.1%), and 98 errors were identified among the 1901 medication orders in the video telemedicine cohort (5.2%). The most common errors were wrong dose (96 of 124 errors [77.4%]) and wrong indication (15 of 124 errors [12.1%]). In treatment-received and per-protocol analyses, the frequencies of medication errors among patients and medication orders were similar to the results in the intention-to-treat analyses and were not significantly different between the telephone and video telemedicine cohorts (Table 2).

**Table 2.** Medication Errors and Types at the Medication Level

Variable	Intention to treat			Treatment received			Per protocol		
	Telephone, errors, No. (%)	Video telemedicine, errors, No. (%)	*P* value	Telephone, errors, No. (%)	Video telemedicine, errors, No. (%)	*P* value	Telephone, errors, No. (%)	Video telemedicine, errors, No. (%)	*P* value
Medication characteristics									
No. of medications ordered	513	1901	NA	1504	910	NA	446	843	NA
At least 1 error present	26 (5.1)	98 (5.2)	.94	73 (4.9)	49 (5.4)	.58	23 (5.2)	48 (5.7)	.91
Error types present									
Wrong dose ordered	20 (3.9)	76 (4.0)	>.99	57 (3.8)	39 (4.3)	.62	20 (4.5)	39 (4.6)	>.99
Too much dose ordered	14 (2.7)	48 (2.5)	.92	35 (2.3)	27 (3.0)	.41	14 (3.1)	27 (3.2)	>.99
Too little dose ordered	6 (1.1)	28 (1.5)	.76	22 (1.4)	12 (1.3)	.91	6 (1.4)	12 (1.4)	>.99
Wrong route ordered^a^	1 (0.2)	3 (0.2)	>.99	0	4 (0.4)	.02	0	3 (0.4)	.56
Wrong dosage form ordered	0	5 (0.3)	.59	5 (0.3)	0	.16	0	0	NA
Error related to patient information^b^	0	4 (0.2)	.59	2 (0.1)	2 (0.2)	.64	0	2 (0.2)	.55
Medication was ordered for the wrong indication	5 (1.0)	10 (0.5)	.33	9 (0.6)	4 (0.5)	>.99	3 (0.7)	4 (0.5)	.69

Abbreviation: NA, not applicable.

^a^ Includes intravenous, per os, and nasogastric.

^b^ Refers to allergy, drug interaction, and kidney or liver disease.

After adjusting for age, severity of illness, and hospital study period, the adjusted odds ratio of experiencing a medication error among those assigned to video telemedicine was 0.86 (95% CI, 0.49-1.52; *P* = .61). The results were similar in the treatment-received and per-protocol analyses (Table 3).

**Table 3.** Adjusted Models of Physician-Related Medication Errors With Random Intercepts for Patient and Originating Emergency Department

Variable	Intention to treat		Treatment received		Per protocol	
	OR (95% CI)	*P* value	OR (95% CI)	*P* value	OR (95% CI)	*P* value
Consultation mode						
Telephone	1 [Reference]	NA	1 [Reference]	NA	1 [Reference]	NA
Video telemedicine	0.86 (0.49-1.52)	.61	1.42 (0.89-2.29)	.14	0.91 (0.44-1.88)	.80
Age, y	1.00 (0.95-1.04)	.87	0.99 (0.95-1.04)	.78	1.03 (0.97-1.10)	.37
Log-transformed Revised Pediatric Emergency Assessment Tool score	0.97 (0.55-1.71)	.93	1.00 (0.57-1.74)	.99	1.09 (0.53-2.25)	.82
Hospital study period						
First 6-mo	1 [Reference]	NA	1 [Reference]	NA	1 [Reference]	NA
Second 6-mo	1.03 (0.57-1.86)	.93	1.11 (0.62-1.99)	.72	0.92 (0.35-2.45)	.87
Third 6-mo	1.07 (0.60-1.91)	.81	1.02 (0.58-1.80)	.94	1.27 (0.56-2.92)	.57
Fourth 6-mo	0.38 (0.19-0.78)	.009	0.38 (0.19-0.76)	.007	0.30 (0.12-0.74)	.009

Abbreviations: NA, not applicable; OR, odds ratio.

## Discussion

In this pragmatic, cluster randomized, unbalanced crossover trial, we did not find a statistically significant difference in the odds of medication errors among critically ill children receiving care in community and rural EDs who received a pediatric critical care consultation by video telemedicine vs telephone. Medication error rates were similar between comparison groups in the unadjusted and adjusted models, including the intention-to-treat, treatment received, and per-protocol analyses. Although other research has suggested that video telemedicine consultations are helpful among this population with respect to practitioner and caregiver experience, triage decisions, and appropriateness of transfer,^11^ these consultations did not impact physician-related medication errors.

In this study, we found that the overall rate of medication errors was similar to those in other studies of EDs that have CPOE^5^ and lower than those demonstrated in older studies and those in EDs without CPOE.^8,9^ We found that the most common type of physician-related medication error was the wrong dose, which has been a well-documented error in children.^4,7,16^ Despite the assistance from CPOEs, dosing errors were common in this study. This may be due to the inaccuracies, rounding errors by the CPOE, or even the discrepancies in acceptable dose by different pharmacologic references.^7,16^

Although research suggests that telemedicine consultations can improve communication and quality of care and reduce unnecessary transfers,^11,17^ the evidence supporting telemedicine to reduce medication errors is limited. One previous study^9^ by our group found that using telemedicine consultations in the ED was associated with decreased physician-related medication errors. However, that study took place in EDs during a time without readily available CPOE. It is possible that errors with telemedicine are now being reduced by electronic health record safety features and CPOE. In addition, the overall incidence of medication errors per patient was substantially higher in our previous study at 1.22 errors per patient,^11^ in comparison to the frequency of 0.125 errors per patient in the current study, further highlighting reduction of errors associated during time periods with CPOE in place. For this study, the lack of a difference in medication errors was affected by the overall low incidence of medication errors, leading to a decrease in study power.

### Limitations

Our study has some limitations. First, the agreement between pharmacist reviewers was only moderate. We determined medication errors from medical records, which meant that we could only identify errors that were documented. Consultations in this study also occurred approximately 2.5 hours after the patient presented to the ED, during which time most medications were already ordered, limiting the opportunity for the telehealth consultation to have a positive impact. Nevertheless, this is the pragmatic nature in which telemedicine consultations are obtained. We also did not collect information on the recommendations that were given by the physician and, therefore, could not identify which consultations explicitly resulted in medication suggestions from the consulting pediatric critical care practitioner. In contrast, studies that focused on telemedicine pharmacy consultations and saw reductions in medication errors may have benefited from recommendations targeted at medications.^18,19,20^ The ability of the emergency medicine and pediatric intensive care unit physicians to deviate from the assigned consultation mode indicates there may be confounding by indication. In this study, there was a larger proportion of deviations in the telemedicine group than in the telephone group, perhaps reflecting workflow difficulties in incorporating telemedicine or resistance in adoption of telemedicine.^21,22^ Although unmeasured confounders and protocol deviations could have contributed to biased results, the previously published baseline characteristics comparing patients who received consultations that adhered to the study group and those whose consultations deviated from the study group intervention were similar.^11^ We also did not identify any significant differences by intention-to-treat, per-protocol, or treatment-received analyses. In addition, despite use of a previously validated medication error instrument, medication errors and their outcomes are somewhat subjective in nature, and the interrater reliability between pharmacists was only moderate, potentially impacting the validity of the findings. It is unlikely that the raters were biased toward 1 consultation or the other because they were blinded. However, the imprecision of this measure may have biased the overall estimate toward the null. In addition, because the use of telemedicine to provide consultations to pediatric patients in remote EDs varies, further research is needed to better understand the impact of these consultations on measures of quality and patient safety.

## Conclusions

In this cluster randomized crossover trial, among a cohort of critically ill children presenting to community and rural EDs, the use of video telemedicine to deliver pediatric critical care consultations was not associated with fewer physician-related medication errors compared with the use of telephones to deliver these consultations. The role of video telemedicine in providing remote acute care continues to emerge. Medication errors may or may not be amenable to improvement through this modality as electronic prescribing and safety protocols are established at more hospitals. Further research is needed to determine what aspects of pediatric acute care consultations are most likely to be improved with video telemedicine.

## Notes

Supplement 1.
Trial Protocol and Statistical Analysis Plan

Supplement 2. **eAppendix.** Medication Error Data Collection Instrument

Supplement 3.
Data Sharing Statement
