# Supplementary material for: Telemedicine vs Telephone Consultations and Medication Prescribing Errors Among Referring Physicians: A Cluster Randomized Crossover Trial
Source: JAMA Netw Open. 2024 Feb 29;7(2):e240275. doi: 10.1001/jamanetworkopen.2024.0275 (PMC10905304; doi:10.1001/jamanetworkopen.2024.0275)
Supplement: Supplement 1. — Trial Protocol and Statistical Analysis Plan [file jamanetwopen-e240275-s001.pdf]

1) **Protocol Title**: Evidence-Based Tele-Emergency Network Grant Program

   Protocol version 7, 7/5/2017

2) **Author of Protocol:** James P. Marcin, MD, MPH
   ☒ **UC Davis Researcher**

3) **IRB Review History:**
   The Dignity Health System sites have already conducted their own IRB review:
   Mercy Medical Center Redding
   Mercy San Juan Hospital

   The Adventist Health sites and all other independent hospitals have already conducted their own internal IRB and/or compliance review:
   Barton Memorial Hospital
   Colusa Medical Center
   Dameron Hospital
   Frank Howard Memorial Hospital
   Lodi Memorial Hospital
   Marshall Medical Center
   Mayers Memorial Hospital
   Oroville Hospital
   Plumas District Hospital
   Sonora Regional Medical Center
   St. Helena Hospital Clearlake
   St. Helena Napa Valley
   Tahoe Forest Hospital
   Ukiah Valley Medical Center

4) **Objectives:** While telemedicine has been increasingly used as a means of providing critical care consultations to children in Emergency Departments (EDs), little is known about the impact that these consultations have on clinical and financial outcomes. We are therefore proposing a 16 center crossover trial to determine how this model of care impacts several clinical outcomes, including cost effectiveness. The objective of this project is to determine the impact of an existing tele-emergency care network on quality of care, appropriateness of care utilization, medication errors, and cost effectiveness compared to telephone consultations from a healthcare system prospective.

   Our first aim is to assess the effect of tele-emergency care on the quality of care provided to seriously ill children. We will evaluate differences in quality of care among children who receive telemedicine consultations compared to telephone consultations using retrospective chart review and a previously validated instrument specifically developed to evaluate the quality of care delivered to children receiving care in an ED.

   Our second aim is to determine the effect of tele-emergency care on the frequency and appropriateness of admission and transfers from rural hospitals. We will evaluate

differences between admission and transfer rates among children who receive telemedicine consultations compared to telephone consultations. Appropriateness of admission and transfer rates will be evaluated using a retrospective chart review process using two severity of illness measures.

Our third aim is to determine the effect of tele-emergency care on the frequency of physician-related medication errors. Medication errors will be compared between children receiving telemedicine consultations and telephone consultations using retrospective chart review and a previously published instrument developed specifically to evaluate medication errors among children receiving care in the ED.

Our fourth aim is to determine the economic efficiency of tele-emergency care. We will compare the economic efficiency of care provided to children receiving telemedicine consultations and telephone consultations using economic evaluations, including cost analysis and cost-effectiveness analysis. The source of data will be the retrospective collection of the number of consultations using the telephone and telemedicine and estimated of costs of care using previously published data.

5) Background: Many rural EDs are not experienced in the care of pediatric patients, particularly those children who are seriously ill. A potential remedy for this problem is to provide rural and/or underserved EDs with pediatric subspecialty support for emergencies using telemedicine. While the standard of care is to provide these consultations by telephone, telemedicine (the use of live, interactive, audiovisual teleconferencing) may be a better means of providing these consultations.

Pediatric critical care telemedicine program at UC Davis*:* The telemedicine program at the University of California, Davis Children's Hospital was one of the first programs to provide telemedicine consultations in emergency and critical care settings for children. As of January 2014, we have conducted more than 200 emergency telemedicine consultations. There are currently 26 hospital EDs in our pediatric tele-emergency network, 16 of which will be participating in this proposed study.

The Pediatric Emergency and Critical Care Telemedicine Program at UC Davis Children's Hospital has a longstanding ability to provide 24/7 pediatric emergency and critical care consultations using either the telephone or telemedicine to all 26 EDs in the network. The type of consultation obtained (telemedicine or telephone) is currently at the discretion of the receiving ED physician and the consulting pediatric critical care physician. The consultations involve live interactive audiovisual communications from the UC Davis physicians to the patient, referring providers (MD/RN/RT), and the parent/guardians of the patient. Typically, approximately 10% of the children that are most seriously ill (triaged to the highest two categories) receive telemedicine consultations while the vast majority receive only telephone or no consultation.

Study design: This will be a retrospective review of prospective data evaluating different outcomes among children who receive telemedicine consultations versus those who receive telephone consultations. All data that will be collected will be obtained retrospectively from

the de-identified medical records of children presenting to the participating ED that meet the entrance criteria. Ultimately, the use of telemedicine and/or telephone will continue to be up to the discretion of the treating ED physician and the consulting pediatric critical care physician. There will be periods of time where one type of consultation will be encouraged per the study protocol, but patients will continue to receive the most appropriate form of care.

6) **Inclusion and Exclusion Criteria**: The study will include de-identified chart review of children up to 14 years of age presenting to participating EDs who received either a telemedicine or telephone consultation with a UC Davis pediatric or neonatal critical care physician.

7) **Number of Subjects:** Up to 600 total consultations, inclusive, from participating sites.

   The overall goal of this project is to evaluate the impact of telemedicine consultations on seriously ill children with regard to several health and economic outcomes. The proposed project will evaluate an existing telemedicine emergency network based out of the UC Davis Children's Hospital in Sacramento, CA. Since its inception, this program has expanded to include 26 EDs, mostly located in rural, underserved Northern California. For the purposes of this study, we will include 16 of the EDs.

8) **Recruitment Methods:** The de-identified medical records of children up to 14 years of age presenting to the participating EDs who received either a telemedicine or telephone consultation with a UC Davis pediatric or neonatal critical care physician during the study period will be included. The existing UC Davis Transfer Center Children's Requests records in EPIC will be used to identify patients meeting the entrance criteria described above from participating hospital EDs during the study period. Qualifying patient encounters will be identified and documented by date, hospital name, patient name and patient date of birth in a password protected excel database and stored on secure, password protected, encrypted University of California computers. The UC Davis project manager will then send requests for corresponding medical charts for qualifying patient encounters to the associated participating hospital personnel via a hospital-specific password protected excel database through secure email or secure fax with the date of encounter, patient name, and patient date of birth. There will not be any other information or patient identifiers on this spreadsheet. Additionally, there will not be any institution name or any research team members name on the document so the document will be entirely blank besides the date of encounter, patient name, and patient date of birth. When partnering hospitals receive the request, their remote site personnel will copy and de-identify PHI prior to sending back to the UC Davis project manager for inclusion in the study database. Prior to UC Davis researchers sending the information request, each remote site PI will sign a form attesting that they will not re-use or re-disclose those PHIs to any other person or entity.

9) **Compensation to Subjects:** None

10) **Study Timelines:** The study project period will take place from 12/15/2014 to 8/31/2018. The final year will include only data analysis.

11) **Study Endpoints**: The end of the projected enrollment period will be 8/31/2017.

12) **Procedures Involved:**
Data collection: The existing UC Davis Transfer Center Children's Requests records in EPIC will be used to identify patients meeting the entrance criteria from participating hospital EDs. Qualifying patient encounters will be documented by date, hospital name, patient name and patient date of birth in a password protected excel database and stored on secure, password protected, encrypted University of California computers. The UC Davis project manager will then send requests for corresponding medical charts for qualifying patient encounters to the associated participating hospital sites via a hospital-specific password protected excel database through secure email or secure fax with the date of encounter, patient name, and patient date of birth. There will not be any other information or patient identifiers on this spreadsheet. Each remote site PI will sign an agreement attesting that they will not re-use or re-disclose those PHIs to any other person or entity.

Medical records will be copied by remote site personnel and de-identified of all patient and hospital identifications. These de-identified medical records will be used to abstract the necessary data to accomplish the objectives of this proposal. The investigators cannot ascertain the identity of the subjects.

The medical charts will be copied at the remote site and no additional data will be collected at University of California Davis Health System (UCDHS). The data is provided to the Principal Investigator after being de-identified, as the method of reporting is a case report form or a medical record that has been both blacked-out of identifiable information and re-copied.

The de-identified charts will be scanned and securely uploaded into REDCap. REDCap is a secure, encrypted, password protected research database available at UC Davis. The charts will then be reviewed to obtain data for our four outcomes: physician experts will review and score the de-identified medical records as to the quality of care using a previously validated instrument; we will determine from the record the patient disposition; pharmacy experts will review and score the de-identified medical records as to the number of medication errors; knowing the disposition of care, we will determine the costs based on standardized average transfer-hospital admission charges. We are therefore collecting several data points: patient disposition, medication errors, and diagnosis.

We will use a novel cluster randomized unbalanced crossover trial design. The first year of the project will be a "ramp-up" period during which the protocol will be re-reviewed during a site visit to the participating EDs and random block assignments will be generated and delivered. The last year will be reserved for data analysis, study closeout, and manuscript preparation. The intermediate 2-year period will be divided into 4 six-month calendar time periods for carrying out the protocol and data collection. For each 6-month period, each ED will have a randomized treatment assignment for pediatric emergency and critical care consultations ("M" for telemedicine and "P" for telephone.) Participating EDs (the unit of randomization) will be stratified into two strata by size of ED and geographical location. EDs

will then be randomized within-strata to one of the four unbalanced (3:1) crossover treatment assignment sequences, each consisting of a 6-month period: PMMM, MPMM, MMPM, or MMMP. During these assigned periods, the type of consultation being assigned will be **strongly encouraged**, but deviating from protocol (i.e., using telephone consultation when randomized to telemedicine, or vice-versa) will be allowed as needed by the physicians. If a protocol arm deviation occurs, then notes explaining the circumstances must be documented. As telephone consultations remain the current standard of care, we anticipate our intervention to be primarily the encouragement of the use of telemedicine (as opposed to the encouragement of the use of telephone).

Regarding randomization, patients will receive the care that is most appropriate for them. The study will not be a true randomization, because the physicians will determine whether or not to use the telephone or telemedicine for the consultation. While we will encourage one modality over the other at different periods of time, the decision is always up to the treating physicians. Therefore, the risk will be minimized for patients.

We will abstract the following variables from eligible patient medical records:

- Demographic information (age, race, gender, insurance type)
- Specialist consultation data (whether a specialist consult was obtained: Yes/No; and if so, which type: Telephone, Telemedicine)
- The variables required for the Pediatric Risk of Admission (PRISA II)
- The variables required for the Pediatric Emergency Assessment Tool (Re-PEAT)
- Disposition data (whether the patient was discharged, admitted or transferred)
- Outcome data for ED (survival, length of stay in ED)
- Outcome data for hospitalized patients (survival, length of stay, ICU utilization)

Phase two of data analyses will include securely sending a subset of our collected de-identified data to the University of Iowa’s Rural Telehealth Research Center for further analysis. The Rural Telehealth Research Center at the University of Iowa (UOI) was hired by the grant administrators at the Health Resources and Services Administration who has funded this grant effort to six grantees. Each of the grantees are asked to send their de-identified data to UOI for analyses to evaluate the impact of tele-emergency programs across the nation. Grantees are asked to submit the following de-identified data variables relevant to the current project:

- ED visit arrival time
- ED exam start time
- ED visit departure time
- ED discharge disposition
- Completion of tele-ED consultation
- Tele-ED consultation start time
- Tele-ED consultation end time
- Tele-ED technical success
- Race

- Sex
- Age (categories include <1, 1-14)
- ICD 10 code
- Reason for visit
- Primary payer
- Billed amount for ED visit

Economic Evaluation: To evaluate the economic efficiency of the tele-emergency network, we will apply standard economic evaluation methods to data collected during the telephone and telemedicine time periods of the randomized block design. Our team has previously conducted similar analyses comparing patients who have received telephone versus telemedicine consultations and our positive findings are currently under peer review at a major medical journal.

Knowing the disposition of the patient, we will assign any of the following costs from external data sources:

1. Cost of ED treatment (this will be the same for all patients): This will be estimated using a National Emergency Department Sample (NEDS).
2. Cost of Hospitalization: This will be estimated using the KID database from the national Healthcare Cost and Utilization Project (HCUP). The average hospital admission costs will be used to estimate hospital admission to a rural, community, and urban hospital for admissions to those hospitals, respectively.
3. Transport costs: If a patient requires transport, we will use previously published estimates for air or ground transportation costs for interfacility transports.

13) Data and Specimen Banking: N/A

14) Data Management and Confidentiality: The existing UC Davis Transfer Center Children's Requests records in EPIC will be used to identify patients meeting the entrance criteria described above from participating hospital EDs during the study period. The Transfer Center Children's Requests records are existing hospital records in EPIC that track all children-specific requests for consultation or transfer to UC Davis from outside hospitals and clinics. Qualifying patient encounters will be identified and documented by date, hospital name, patient name and patient date of birth in a password protected excel database and stored on secure, password protected, encrypted University of California computers. The UC Davis project manager will then send requests for corresponding medical charts for qualifying patient encounters to the associated participating hospital personnel via a hospital-specific password protected excel database through secure email or secure fax with the date of encounter, patient name, and date of birth. There will not be any other information or identifying information on this spreadsheet.

Once qualifying patient encounters are identified, medical charts will be copied and de-identified by remote hospital site personnel. All data collected from each of the participating hospitals will be collected confidentially so that no patient identifiers or links to patient and hospital identifiers will be contained in the research records. This

will be accomplished by "blacking out" all patient and hospital identifiers permanently with a black marker by remote site personnel photocopying the medical records. A unique identification number will be assigned to each of a patient's data. Each site will be responsible for holding the key with the unique identifier and is prohibited from releasing the key to the study investigators under any circumstance.

The blacked-out copies will be re-copied to ensure that text cannot be read through the marker. These de-identified, re-copied medical records will be either collected in-person by the study personnel or will be sent to the study investigator by certified mail. These copied medical records (without patient or hospital identifiers) will be used to fulfill the objectives of the grant. The same de-identified medical records will be used to obtain the data necessary for quality of care, severity of illness measures, medication errors, and patient disposition. Since existing records will be used and all patient and hospital identifiers will be eliminated, there are no anticipated risks to subjects whose records will be reviewed.

All the medical records included in the analyses will be photocopied at the remote sites. These records will be de-identified prior to leaving the Medical Records Department at the participating institutions. The de-identified medical record will be stored in a locked office which resides in a secured building within UC Davis Children's Hospital. All of the data that pertains to the patient and data for quality measures will be abstracted from the de-identified medical record and entered into a secure password-protected database on a single password-protected personal desktop computer in a locked office at UC Davis Children's Hospital. All study personnel will be trained through an in-person training session before the start of the project on the data abstraction, collection and data entry process. All data will be managed, cleaned, and analyzed at UC Davis Children's Hospital. All study personnel, software, computers and research resources required to conduct this study will be supported from this grant except for the pediatric subspecialist. The investigators and staff are fully committed to the security and confidentiality of all data collected from the participating hospitals. All study personnel involved in this proposed project have received Human Subjects Protection and HIPAA education.

The data from this program will be shared with only statistical consultants and an economist, who will ensure that the data also be on a single password-protected desktop computer in a locked office in a secured building.

15) **Provisions to Monitor the Data to Ensure the Safety of Subjects**: Research does not involve more than Minimal Risk to subjects.

16) **Withdrawal of Subjects:** This study only involves the retrospective review of medical records, so withdrawal of subjects is not applicable.

17) **Risks to Subjects:** A breach of confidentiality, which is always a risk when accessing medical records, is the only risk to subjects. The risk to subjects is considered minimal as we do not anticipate loss to individual confidentiality, as we will be using only coded medical

records and surveys. All medical records that will be used in this study will have all patient identifiers and links to patient identifiers removed prior to leaving the Medical Records Department at the participating hospitals. All of the data that pertains to the subjects' utilization data, admission and transfer rates, and cost benefit data, including the anonymous records, will be stored in the secure locked office building within the UC Davis Medical Center.

18) **Potential Benefits to Subjects**: There is no direct benefit to subjects.

19) **Vulnerable Populations:** This study will involve the review of the medical records of children. All other vulnerable populations will be excluded.

20) **Multi-Site Research:** This study is a chart review study; the charts are collected and de-identified at the participating sites and stored at UC Davis. We will use email as the primary mode of communication with all the participating sites and each site will be trained in the study protocol. All required approvals will be obtained from each site. Any modification to the study protocol will be immediately communicated to each site through email.

21) **Community-Based Participatory Research:** None

22) **Sharing of Results with Subjects:** None

23) **Setting:** The chart abstraction of the de-identified medical records, data entry, management and data analysis will be conducted at UC Davis.

24) **Resources Available:** University computers (encrypted, secure and password protected) will be used for data collection and analyses. Medical records will be stored in a locked cabinet in a locked office in a secure building (the Center for Health and Technology).

    Participating rural hospitals will receive financial support to assist with administrative duties of the project (e.g., photocopying). Additionally, administrative and IRB review fees will be paid to hospitals as necessary.

    Designated staff will include Principal Investigator, two co-investigators, a health economist, a statistician, and a program manager/research analyst. The Principal Investigator has extensive experience, both clinically and operationally, with telemedicine and the analysis of telemedicine data. This project extends previous federally funded research of the UC Davis Pediatric tele-emergency network.

    The co-investigators, both highly accomplished research investigators and clinicians, will provide their knowledge and expertise to the project. The health economist and statistician will assist with data analysis. The research analyst will manage communication with the participating hospitals and assist the Principal Investigator and co-investigator with research and project goals. All staff is qualified to perform the proposed research.

25) **Prior Approvals**: Approvals will be obtained from partner sites.

26) **Provisions to Protect the Privacy Interests of Subjects:** N/A

27) **Compensation for Research-Related Injury:** N/A

28) **Economic Burden to Subjects:** Subjects will not be responsible for any further economic burden.

29) **Consent Process:** Consent will not be obtained. The study is a chart review study.

30) **Process to Document Consent in Writing:** Research presents no more than minimal risk of harm to subjects.

31) **Drugs or Devices:** None
