# Supplementary material for: Telemedicine vs Telephone Consultations and Medication Prescribing Errors Among Referring Physicians: A Cluster Randomized Crossover Trial
Source: JAMA Netw Open. 2024 Feb 29;7(2):e240275. doi: 10.1001/jamanetworkopen.2024.0275 (PMC10905304; doi:10.1001/jamanetworkopen.2024.0275)
Supplement: Supplement 2. — eAppendix. Medication Error Data Collection Instrument [file jamanetwopen-e240275-s002.pdf]

Supplemental Online Content

Marcin JP, Lieng MK, Mouzoon J, et al. Telemedicine vs telephone consultations and medication prescribing errors among referring physicians: a cluster-randomized crossover trial. *JAMA Netw Open*. 2024;7(3):e240275. doi:10.1001/jamanetworkopen.2024.0275

eAppendix. Medication Error Data Collection Instrument

This supplemental material has been provided by the authors to give readers additional information about their work.

© 2024 Marcin JP et al. *JAMA Network Open.*

eAppendix: Medication Error Data Collection Instrument.

Physician-related medication error: Defined as: Wrong dose; Wrong or inappropriate drug for condition; Wrong route; and Wrong dosage form.

- ☐ Medication given but not ordered
- ☐ Medication ordered but not given
- ☐ Wrong drug given from what was ordered
- ☐ Wrong dose
  - ☐ Too much
  - ☐ Too little
- ☐ Wrong or inappropriate drug ordered for condition (your opinion)
- ☐ Wrong administration technique (eg, rate)
- ☐ Wrong route (IV, PO, PR, SQ, IM)
- ☐ Wrong dosage form
- ☐ Wrong time (> 30 min from order)
- ☐ Error related to patient information (e.g., allergy, drug interaction, renal or hepatic disease)
- ☐ Other (describe): ________________________________
  ____

Administration Technique: IV – Intravenous route, PO – Oral route, SQ – Subcutaneous route, IM – Intra-muscular route.

1. Antipyretics	+/- 20%
2. Antibiotics	+/- 25%
3. Steroids (e.g., methyl prednisone, prednisone, prednisolone)	+/- 25 %
4. Narcotics (e.g., fentanyl, morphine, codeine)	+/- 25%
5. Anxiolytics (e.g., lorazepam, midazolam, diazepam)	+/- 20%
6. Code medications (e.g., boluses of atropine, epinephrine, lidocaine, adenosine, HCO3)	+/- 10%
7. Anesthetics (e.g., ketamine, etomidate, thiopental, pentobarbital)	+/- 10%
8. Paralytics (e.g., succinyl choline, vecuronium, rocuronium)	+/- 20%
9. Inotropes, Vasoactives, Cardiac medications (e.g., epinephrine, lidocaine, dopamine)	+/- 10%
10. Insulin drip	+/- 10%
11. Dextrose and electrolytes (e.g., potassium, calcium)	+/- 20%
12. Anticonvulsants (e.g., phenytoin, lorazepam)	+/- 20%

* “Acceptable ranges” were determined to be those doses above and below the upper and lower limits of the ranges published by Lexi-Comp’s Pediatric Dosage Handbook[12]

© 2024 Marcin JP et al. *JAMA Network Open.*
