# Supplementary material for: Telemedicine vs Telephone Consultations and Medication Prescribing Errors Among Referring Physicians: A Cluster Randomized Crossover Trial
Source: JAMA Netw Open. 2024 Feb 29;7(2):e240275. doi: 10.1001/jamanetworkopen.2024.0275 (PMC10905304; doi:10.1001/jamanetworkopen.2024.0275)
Supplement: Supplement 3. — Data Sharing Statement [file jamanetwopen-e240275-s003.pdf]

Data Sharing Statement

Marcin. Telemedicine vs Telephone Consultations and Medication Prescribing Errors Among Referring Physicians. *JAMA Netw Open*. Published March 01, 2024.
doi:10.1001/jamanetworkopen.2024.0275

Data
Data available: Yes
Data types: Deidentified participant data, Other (please specify)
Additional Information: Upon Request
How to access data: Upon Request: jpmarcin@ucdavis.edu
When available: With publication

Supporting Documents
Document types: None

Additional Information
Who can access the data: Made available to researchers upon request
Types of analyses: for any purpose
Mechanisms of data availability: Upon Request: jpmarcin@ucdavis.edu
